# Reading habits of medical practitioners: Young doctors in Pakistan, a case study

**DOI:** 10.1016/j.jtumed.2022.04.007

**Published:** 2022-05-11

**Authors:** Muhammad Ali Raza, Faizah Mohamad Nor, Rashid Mehmood

**Affiliations:** a Language Academy, Faculty of Social Sciences and Humanities, Universiti Teknologi Malaysia, Johor, Malaysia; b English Language Institute, Jazan University, Jazan, KSA; c Department of Mathematics, University of Hafr Al Batin, Hafr Al Batin, KSA

**Keywords:** التعليم الطبي, الأطباء, عادات القراءة, تفضيلات القراءة, باكستان, المسوحات والاستبيانات, Online reading, Pakistani physicians, Print reading, Reading habits, Reading medical journals, Reading preferences

## Abstract

**Objective:**

This study attempted to investigate the reading preferences and habits among young Pakistani medical doctors. The reading time, preferred source of information, preferred medical journals, and ways of reading medical journals were explored.

**Methods:**

A survey approach was used for data collection. The study participants were young medical professionals in Pakistan. An online survey was sent to more than 300 individuals through various physicians and their professional groups/bodies. A total of 155 responded to the questionnaire, and 128 of the questionnaires were considered worthy of data analysis.

**Results:**

Among respondents, 40% read printed journals, 49% read online journals, 60% read case reports, and 55% read newspapers for 1–5 h per week. Continuing medical education was the preferred source of information, and the Pakistan Journal of Cardiology & Thoracic Surgery was the preferred medical journal. Reading the abstract and the conclusion was the preferred way of reading journal articles.

**Conclusion:**

Young physicians are enthusiastic in participating in research activities and spending time gaining updated information. Physicians read articles methodically. Online sources of information are preferred over printed sources.

## الملخص

**أهداف البحث:**

تحاول هذه الدراسة التحقق من تفضيلات القراءة وعادات الأطباء الباكستانيين الشباب. تستكشف الدراسة وقت القراءة، والمصدر المفضل للمعلومات، والمجلات الطبية المفضلة، وطرق قراءة المجلات الطبية.

**طرق البحث:**

في الدراسة الحالية، تم استخدام نهج المسح لجمع البيانات. كان مجتمع الدراسة من الأطباء الشباب الذين يعملون في باكستان. تم إرسال استبانة عبر الإنترنت إلى أكثر من 300 فرد من خلال مختلف الأطباء ومجموعاتهم / هيئاتهم المهنية. رد ما مجموعه 155 طبيبا على الاستبانة، وكانت 128 إجابة فقط منها جديرة بتحليل البيانات.

**النتائج:**

من 1 إلى 5 ساعات هو الوقت الذي يقضيه 40٪ من المستجيبين في قراءة المجلات المطبوعة. 49٪ أمضوا نفس القدر من الوقت في قراءة المجلات على الإنترنت، و 60٪ في قراءة الأبحاث حول الحالات، و 55٪ في قراءة الصحف. كان "التعليم الطبي المستمر" هو المصدر المفضل للمعلومات. كما كانت المجلة الباكستانية لأمراض القلب وجراحة الصدر هي المجلة الطبية المفضلة. وكانت قراءة الملخص والاستنتاج هي الطريقة المفضلة لقراءة مقالات المجلات.

**الاستنتاجات:**

الأطباء الشباب في باكستان متحمسون للمشاركة في الأنشطة البحثية وقضاء الوقت في تحديث المعلومات. يقرأ الأطباء الشباب المقالات بشكل منهجي. تُفضل مصادر المعلومات عبر الإنترنت على المصادر المطبوعة.

## Introduction

In the absence of regular updating, physicians' knowledge and skills are prone to becoming obsolete over time.^1^ Schwartz^2^ has suggested that reading aids in exploring ideas on best practices in medical care. Reading multiple authors provides an opportunity to view and analyze their perspectives, and improves understanding of the “ponderables” (p. 354). Khan et al.^3^ have suggested that developing a reading habit is crucial for successful medical practice. Online reading resources enhance physicians' skills.^4^ Healthcare competencies required of physicians are developed through critical reading and in-depth analysis of the latest research and published experiences.^5^ Chettri and Rout^6^ have argued that a reading habit is essential “for the acquisition of new knowledge and skills, for gaining information through media, especially newspapers, books, radio, television, and the computers” (p. 13).

Little attention has been paid to studying the reading habits of young physicians in Pakistan, particularly through considering print reading and online reading habits as separate entities. For example, Fafard and Snell,^7^ Saint et al.,^8^ Schein et al.,^9^ Leff and Harper,^10^ Soliman and Neel,^11^ and Amani and Jaffar^12^ have studied reading habits in terms of time spent on reading journal articles, preferred source of information, case reports, and journal subscriptions.

In addition to medical journals, newspapers have roles in the professional lives of physicians that cannot be overlooked. Rapid dissemination of information through newspapers has been demonstrated to be helpful to medical professionals and the concerned authorities. For example, most Vietnamese medical professionals, medical students, and healthcare workers acquire most of their information on COVID-19 through online newspapers, social media, and the Internet.^13^ The health ministry in Bangladesh uses newspapers along with other platforms for timely and effective dissemination of information on COVID-19.^14^ Buonanno and Puca^15^ have presented a technique of using obituaries published in newspapers in Italy to estimate and forecast the mortality rates due to the COVID-19 outbreak on a daily basis. Nguyen and Vu^16^ have also collected data on the COVID-19 outbreak in Vietnam.

The information received through newspapers (both online and printed) is important in helping medical professionals and decision-makers improve healthcare services. For example, Huda et al.^17^ have found that negligence and mishandling of patients with COVID-19 in hospitals in Bangladesh were reported in newspapers. Therefore, they have suggested that “The stakeholders should take appropriate measures so that patient confidence in the health-care system can be restored.”^17^ Online newspapers can serve as effective tools for disseminating information on COVID-19. Moreover, printed newspapers can be used to elicit behavioral changes.^13^ Public health officials often use newspapers and social media to keep abreast of the information released by government agencies, which may be crucial to emergency responses.^18^ These platforms serve as additional data sources for detecting disease outbreaks and cases via the public health surveillance systems.^19^

### Problem statement

Newspapers (online and printed) play major roles in disseminating information associated with disease and viruses to the masses, physicians, and healthcare professionals. Newspaper information is not considered completely reliable for medical professionals, because of the absence of a peer review process. However, immediate and mass delivery of information is a major advantage of newspapers. Moreover, Jamshed^20^ contends that the information on COVID-19 in newspapers is more reliable than that in social media.

A renowned cardiothoracic surgeon in Lahore, Pakistan, has informed the authors that, in addition to medical journals, newspapers (particularly online and sometimes printed) are indispensable for physicians. Newspapers are a tool for the fastest delivery of the latest information on diseases, viruses, infections, epidemics, and pandemics. For example, physicians use information from newspapers regarding the spread of the COVID-19 pandemic to develop/improve/adapt plans to fight against the virus. Newspapers also disseminate crucial medical health-related information to the masses and healthcare professionals (Liman^21^; Wilbur^22^; Tran et al.^13^). Therefore, the roles of newspapers in healthcare professionals' reading habits should not be ignored. However, because newspapers are not the primary source of information for physicians, limited focus has been placed the role of newspapers. Hence, printed and online newspapers have not been considered as separate entities.

### Contribution of this study

The established presence and role of medical journals and newspapers associated with reading habits should not be overlooked. Therefore, newspapers (including online and printed) were considered as a variable in this study of the reading habits of healthcare practitioners. The study also attempted to discover the reading preferences among Pakistani healthcare practitioners, to determine the preferred sources of information, ways of reading journal articles, and preferred medical journals. Moreover, the time spent reading printed journals, online journals, and case reports was examined. Finally, the study also determined the time spent reading newspapers (both online and printed).

### Research questions

1. How much time do physicians spend reading?
2. What are physicians' preferred sources of information?
3. What ways of reading articles are used by physicians?
4. Which medical journals are preferred by physicians?

## Materials and Methods

### Study setting

The study was conducted between January and November 2020 among Pakistani physicians serving in Pakistan. An online survey was used to collect responses from young physicians.

### Population and sampling

The study population was young Pakistani physicians serving the country in urban and rural settings. The sample^23^ included respondents in the age range of 21–35, genders, specialties (i.e., primary and secondary healthcare), and type of service (i.e., teaching, practice, or teaching and practice).

The sampling technique used for this study was clustered sampling.^24^^,^^25^ Email invitations with links to the survey form were distributed to the participants. The participating physicians completed the surveys during their leisure time.

### Study instrument

For measuring reading attitude and preferences, this study adapted the Adult Survey of Reading Attitude (ASRA) used by Karim and Hasan^26^ and Smith.^27^ The survey responses were on a five-point Likert scale (with 5 indicating the most preferred and 1 indicating the least preferred) to gauge the reading preferences and preferred sources of information in three questions. The questionnaire consisted of four sections.

The first section covered personal and demographic information. This section included questions regarding age, gender, geographic area (rural/urban), type of practice, and specialty. The question on gender comprised three options and a “rather not say” option. The question regarding the geographic area of work provided two options: rural or urban. The questions on the type of practice provided three options: teaching, practice, or both. Questions regarding the specialty provided two options: primary healthcare and secondary healthcare.

The second section comprised questions about reading habits, i.e., the number of hours spent reading printed journals, online journals, case reports, and newspapers. Four questions pertained to the time spent on reading (printed journals, online journals, case reports, and printed and online newspapers). The questions provided five options: 0 h per week, 1–5 h per week, 6–10 h per week, 11–20 h per week, and more than 20 h per week.

The third section on reading preferences included questions on the preferred source of information and preferred medical journal. The question on the preferred source of information comprised ten subparts. The options to choose the preferred source of information consisted of a Likert scale as follows: most preferred = 5, more preferred = 4, preferred = 3, less preferred = 2, and least preferred = 1.

The fourth section sought responses regarding the mode of reading journal articles. This question provided seven combinations as options: reading the abstract, introduction, discussion, conclusion, references, or the whole article; scanning for the desired information; and taking notes while reading journal articles. The options to choose the preferred mode of reading consisted of a Likert scale as follows: most preferred = 5, more preferred = 4, preferred = 3, less preferred = 2, and least preferred = 1.

### Statistical methods

Data analysis was conducted in version 16 of Statistical Package for Social Sciences (SPSS). Descriptive analysis was conducted in the form of the median for each variable. Microsoft Excel was used to create visual representations of the findings in bar charts.

## Results

### Response rate

A total of 155 questionnaires were received, and 128 responses with complete essential information were deemed suitable for data analysis. Thus 82.6% of questionnaires were considered in the data analysis.

### Respondents' characteristics

The distribution of participants in terms of gender was as follows. Sixty-three participants were women (49%), whereas 60 (47%) were men, and 5 (4%) did not want to disclose their gender. One hundred and six (83%) respondents served in urban areas, and 22 (17%) served in rural settings. The types of service were: teaching (14; 11%); practice (95; 74%); and practice and teaching (19; 15%). The specialties were primary healthcare (91; 71%); and secondary healthcare (37; 29%).

### Reading time

Section two addressed the time spent reading materials including printed journals, online journals, case reports, and newspapers (including online and printed newspapers).

**Figure 1:**
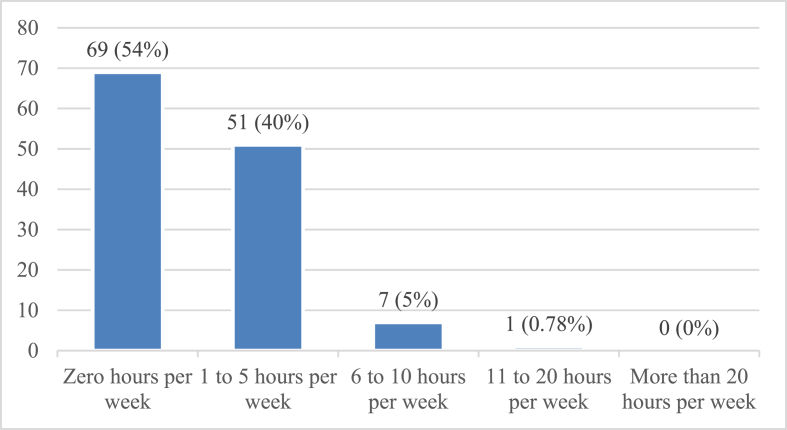
Frequency of respondents reading printed journals.

Figure 1 displays the time spent reading printed journals. Most young Pakistani physicians reported spending 0 h per week reading printed journals (69; 54%). A total of 51 (40%) respondents reported spending 1–5 h reading printed journals, and only 7 (5%) respondents claimed to read printed journals for 6–10 h per week. Finally, only 1 respondent (1%) reported reading printed journals for 11–20 h per week. No respondents indicated spending more than 20 h reading. Therefore, 59 (46%) physicians were inferred to read printed journals (including data on reading 1–5, 6–10, 11–20, and 20+ hours per week), whereas 69 (54%) did not spend any time reading printed articles.

**Figure 2:**
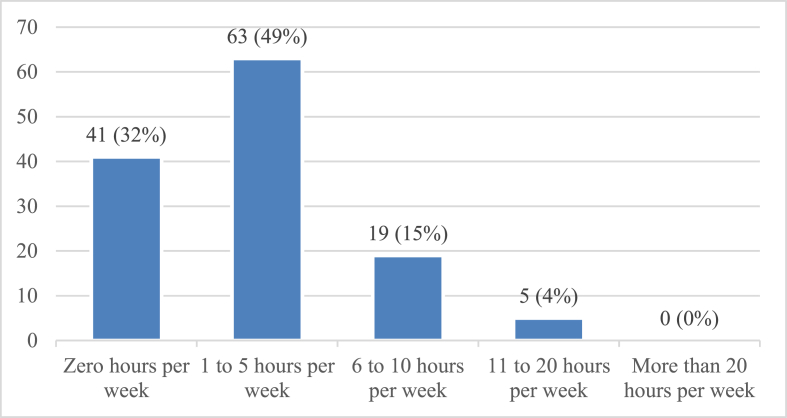
Frequency of respondents reading online journals.

Figure 2 shows the time spent reading online journals. Most physicians reported reading for 1–5 h every week. The frequency of the responses was 63, representing 49%. The second value was for 0 h spent reading online journals, with a frequency of 41, representing 32%. A total of 19 participants (15%) read online journals 6–10 h per week. Five physicians (4%) spent 11–20 h reading online journals per week. However, as with reading of printed journals, no participants indicated reading online journals for more than 20 h. From the data, 87 (68%) physicians were inferred to read online journals (including data on reading 1–5, 6–10, 11–20, and 20+ hours per week), whereas 41 (32%) did not spend any time reading online journals.

**Figure 3:**
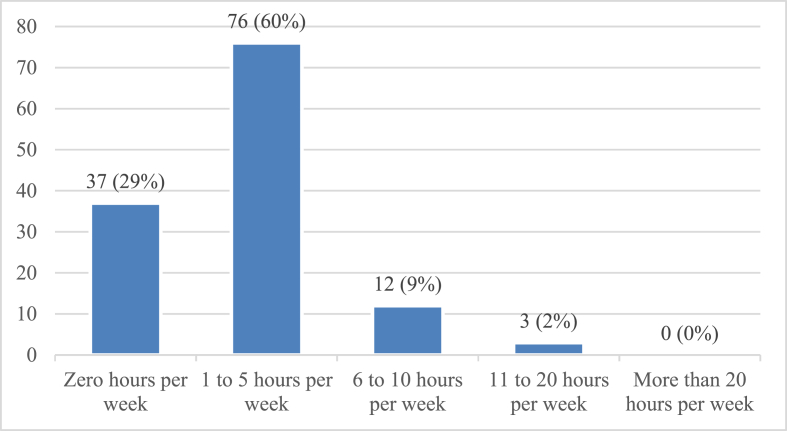
Frequency of respondents reading case reports.

Figure 3 represents the time spent reading case reports. A clear majority (76; 60%) reported spending 1–5 h per week reading case reports. Twelve physicians (9%) performed weekly reading of case reports for 6–10 h. Only three (2%) physicians spent 11–20 h per week reading case reports. No participants indicated a weekly reading time for case reports of more than 20 h. Interestingly, 37 (29%) physicians did not spend any time reading case reports. On the basis of these findings, 91 (71%) physicians were inferred to read case reports (including data on reading 1–5, 6–10, 11–20, and 20+ hours per week), whereas 37 (29%) did not spend any time reading case reports.

**Figure 4:**
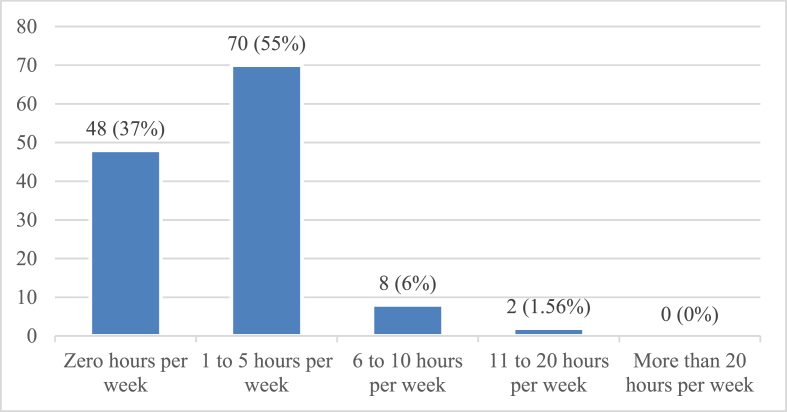
Frequency of respondents reading newspapers.

Figure 4 sums the time spent the reading newspapers (online and printed newspapers together). A total of 70 (55%) physicians reported reading newspapers 1–5 h every week. Interestingly, 48 (37%) spent not even a single hour per week reading newspapers. A total of eight (6%) and two (2%) physicians had 6–10 weekly reading hours and 11–20 weekly reading hours, respectively. No respondents indicated reading newspapers for more than 20 h per week. Thus, 80 (62.5%) physicians were inferred to read newspapers (including data on reading 1–5, 6–10, 11–20, and 20+ hours per week), whereas 48 (37.5%) did not spend any time reading newspapers. Consequently, Pakistani physicians were quite interested in reading newspapers.

**Figure 5:**
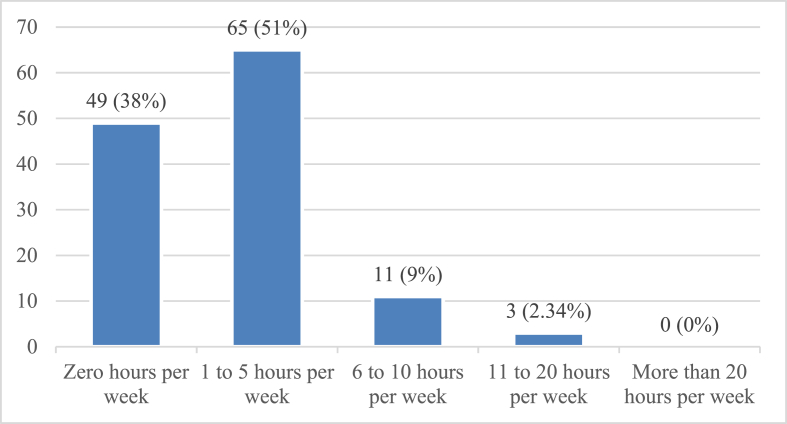
Total reading time, including printed journals, online journals, case reports, and newspapers.

In Figure 5, the total time that Pakistani physicians spent reading sources including printed journals, online journals, case reports, and newspapers is shown. The data revealed that 65 (51%) of physicians spent 1–5 h on reading, 49 (38%) physicians spent no time on reading, 11 physicians (9%) spent 1–6 h on reading, and only 3 physicians (2%) spent 11–20 h on reading these sources per week. It may be inferred that most of the physicians reported spending considerable time on reading newspapers. This suggests that newspapers are relevant for the Pakistani physicians.

### Preferred sources of information

The third section addressed the preferred sources of information and the preferred medical journal.

Table 1 shows responses to the question regarding the preferred sources of information. Continuing medical education was the most preferred information source (115 responses, median score 4). The second most preferred source of information was senior medical colleagues (114 responses, median score 4). The third most preferred source was medical literature/libraries (112 responses, median score 4). The fourth most preferred source was medical websites (109 responses, median score 4). The fifth most preferred source was videos/television (89 responses, median score 4).

**Table 1:** Preferred sources of information.

Preferred source of information	Median Likert score	No. of responses
Senior medical colleagues	4	114
Medical literature/library	4	112
Continuing medical education and courses	4	115
Pharma. Reps	3	108
Audio tapes/files	3.5	88
Videos/television	4	89
Medical websites	4	109
Social media	3	106
Printed newspapers	3	105
Online newspapers	3	105

The physicians reported using continuing medical education, senior medical colleagues, medical literature/libraries, medical websites, and videos/television to gather information in their fields. Interestingly, the physicians indicated that the above five sources were more preferred, represented by a score of 4.

In the next part of the questionnaire, the study sought responses regarding the preferred medical journal.

Table 2 reveals that the Pakistan Journal of Cardiology and Thoracic Surgery (PJCTS), British Medical Journal (BMJ), American Journal of Medicine (AJM), and Journal of the American Medical Association (JAMA) were the more preferred journals among Pakistani physicians. PJCTS had an 89 responses and a median score of 4; BMJ 85 responses, median score 4; AJM 85 responses, median score 4; and JAMA had 82 responses, median score 4.

**Table 2:** Preferred medical journals.

Preferred medical journal	Median Likert score	No. of responses
AJM (American Journal of Medicine)	4	85
JAMA (Journal of the American Medical Association)	4	82
SMJ (Southern Medical Journal)	3	78
Lancet	3	80
AIM (Annals of Internal Medicine)	3	79
BMJ (British Medical Journal)	4	85
NEJM (New England Journal of Medicine)	3	80
HP (Hospital Practice)	3	80
PJMR (Pakistan Journal of Medical Research)	3	94
PJP (Pakistan Journal of Pharmacology)	3	87
PAFMJ (Pakistan Armed Forces Medical Journal)	3	91
PJCTS (Pakistan Journal of Cardiology & Thoracic Surgery)	4	89
JPMA (Journal of the Pakistan Medical Association)	3	95
Other	3	51

### Way of reading articles

The fourth section investigated how physicians read journal articles.

As shown in Table 3, reading the abstract and the conclusion was the most frequently used way of reading articles (108 responses with a median score of 4). Scanning the article for the desired information was the second most preferred way of reading articles (95 responses, median score 4).

**Table 3:** Ways of reading articles.

Way of reading articles	Median Likert score	No. of responses
Reading abstract and conclusion	4	108
Reading abstract, introduction, and conclusion	3	91
Reading abstract, introduction, discussion, and conclusion	3	88
Reading abstract, introduction, discussion, conclusion, and references	3	76
Reading the whole article	3	78
Scanning articles to get information	4	95
Taking notes about important information	3	85

## Discussion

The participants were almost equally divided by gender: women made up 49% and men made up 47%. Most (71%) of the physicians served in primary healthcare. A clear majority of Pakistani physicians, i.e., 51% (65 physicians), had a total reading time of 1–5 h per week (including time spent reading printed journals, online journals, case reports, and newspapers). Khaliq et al.^28^ have studied reading in terms of the number of articles read each week and have found that 10.9% of physicians read 6–10 h per week. Nevertheless, the findings of the present study correspond to the data gathered by Khaliq et al. Several other studies have found similar results. In a study by Fafard and Snell,^7^ house staff reported 8.7 h of weekly reading time. Saint et al.^8^ have reported an average of 4.4 weekly hours of journal article reading time. The surgeons in a study by Schein et al.^9^ spent 14 h each month reading medical literature. Malaysian university students have been found to spend 9 h reading per week, according to Karim and Hasan.^26^

Overall, the most common reading activity among Pakistani physicians was reading case reports (71% of the participants). Among the physicians with 1–5 h of weekly reading time, 60% read case reports. This finding confirms those reported by Fafard and Snell,^7^ wherein the house staff reported spending 8.7 h per week reading case reports. However, 29% of physicians in that study did not spend any time reading case reports, because of a heavy patient load, insufficient time, not having personal digital assistants, and unreliable availability of Internet service.^28^

The participants spent more time reading online journals than printed journals. A total of 68% (87) of physicians read online journals, whereas 54% (69) read printed journals. The results corresponded to the findings of Amani and Jaﬀar,^12^ in which most respondents among medical practitioners in Bahrain (57.4%) preferred online journals to printed journals (42.6%). In addition, in a study by Leff and Harper,^10^ 81% of the respondents read online journals articles. In Khaliq et al.,^28^ Pakistani physicians preferred e-journals (50.6%) to printed journals (32%). Soliman and Neel^11^ have reported that Saudi medical students prefer online reading resources, because they find them most useful.

Our findings also revealed that newspapers were relevant for Pakistani physicians: 62% of the respondents reported reading newspapers. These findings are consistent with those reported by Karim and Hasan,^26^ in which 74% of Malaysian university students spent a considerable amount of time reading newspapers and other materials. The researchers' interviews with physicians also confirmed that physicians read newspapers to keep up to date regarding healthcare issues and the success of departmental efforts.

The next finding concerned the preferred sources of information. Continuing medical education was the most favored information source among Pakistani physicians, with 115 responses. These findings differ from those of Khaliq et al.,^28^ in which 71.4% of Pakistani physicians preferred medical books as a source of information. In Schein et al.,^9^ continuing medical education ranked third among the preferred information sources. Moreover, medical literature was the choice of most health professionals in a study by Stinson and Mueller,^29^ with a mean value of 2.5. At King Saud College of Medicine, KSA, 93% of students have reported using medical pocketbooks as a preferred source of information.^11^ The online source UpToDate® received the maximum rating among medical students in a study by Leff and Harper.^10^ The data revealed that preferred information sources varied according to factors including demography. Hence, every country might have a different set of preferred information sources.

PJCTS was the most commonly used journal in this study, with a median score of 4 and 89 responses. In a study by Khaliq et al.,^28^ the Journal of the Pakistan Medical Association (JPMA) was the most commonly used journal, with an average of 63.2%. Aslam and Waheed^30^ have found that JPMA was commonly used among the students of two private medical colleges in Sindh, Pakistan, partly because the journal provides a students' corner to publish students' articles. One common aspect among Khaliq et al.,^28^ Aslam and Waheed,^30^ and this study was that all three studies found that Pakistani journals were the most commonly used journals among Pakistani physicians.

The data on the preferred medical journal revealed that the preferred foreign medical journal was the BMJ, with a median score of 4 and 85, whereas the New England Journal of Medicine was the most frequently read journal in Fafard and Snell.^7^ Saint et al.^8^ have reported that 72% of the respondents read Annals of Internal Medicine. Annals of Surgery was the most prominent journal among the frequently read journals in Schein et al.^9^ The physicians' favorite journal in Bahrain, as reported by Amani and Jaﬀar,^12^ was the AAFP Family Medicine Journal. All of the above studies have reported different foreign journals being commonly used among medical professionals. Thus, the preference for medical journals varies with demography.

In this study, most of the Pakistani physicians read only the abstracts and conclusions, with a median score of 4 and 108 responses. This finding partially aligns with those of Khaliq et al.,^28^ who have found that 51% of Pakistani physicians read only the abstracts of articles. The respondents in Saint et al.^8^ reported reading the abstract of 63% of articles. Ejaz et al.^31^ have found that new medical graduates in Karachi (Pakistan) are interested in research, provided that they receive encouragement. The methodical reading of articles in this study is also in line with findings reported by Subramanyam.^32^

## Conclusion

The young physicians practicing in urban areas of Pakistan were usually enthusiastic about participation in research activities. The physicians preferred reading case reports and online journals. The physicians perceived reading case reports as crucial for their practice. This finding highlights that students of medicine must devote time to reading case reports, to enhance their competency and ensure their professional progress. Printed journals were least preferred among the physicians, whereas continuing medical education was a preferred source of information. This information should help medical governing bodies, such as the Pakistan Medical Commission, revise and improve curricula and professional development programs. PJCTS was the preferred Pakistani journal, and the preferred foreign journal was the BMJ. The information regarding preferred medical journals (foreign and local) should help policymakers take measures to motivate physicians to read more diverse range of journals. Most of the Pakistani physicians read only the abstracts and conclusions of most articles. Thus, students of medicine must develop a habit of smart reading. Newspapers were found to be relevant to the physicians, most of whom reported spending some hours reading newspapers weekly. In addition to the information retrieved through the additional data sources such as newspapers, the medical authorities will be able to make medical decisions. Pharmaceutical firms will be able to use newspapers to improve the acceptability of their products.

As a whole, the findings should help medical practitioners and house staff develop reading habits, knowledge, and awareness. Academic development of professions in medicine could use the information retrieved from this research to support the design, development, and execution of mid-career academic development programs. Pharmaceutical firms and businesses in the field could use this information for planning and promoting their business activities and products. Medical librarians could use the information to improve their services. Librarians could further their understanding of their users' reading habits and requirements. Hospital managers could develop more effective working schedules in light of the findings, so that their staff can have more time to read.

### Scope of future research

Future research should be conducted in the field of retention of acquired knowledge and skills, evaluation of comprehension, quality of reading, and the efficiency of reading medical newspapers and medical journal articles. The presence, acceptance, roles, and efficacy of social media (including Facebook, Twitter, Instagram, LinkedIn, Snapchat, Tumblr, and Pinterest) in the field of medicine are also worthy of study.

### Limitations

Most physicians responding to the research participation invitation were younger than 35 years of age. The technique of self-reporting has inherent limitations. The study was not funded by any government or non-governmental organization; therefore, the participants were not paid for their contributions.

## Source of funding

This research did not receive any specific grant from funding agencies in the public, commercial, or not-for-profit sectors.

## Conflict of interest

The authors have no conflict of interest to declare.

## Ethical approval

The authors declare that no experiments on animals or humans were conducted during this study. The procedures involved in this study were online questionnaires and interviews. The participants were told that there were no monetary benefits for participation in this study and that they could withdraw their participation at any time. The identities of respondents were coded to maintain privacy and anonymity. The participating physicians were informed of the information sought and the ways of using the retrieved information.

## Authors contributions

FMN supervised the work, developed the study design and methods, improved the data analysis, and reviewed and edited the article. MAR conceptualized the study, collected data, analyzed and interpreted data, and wrote and edited the manuscript. RM was involved in conceptualization of the study, improvement of the statistical analysis, findings, and correction of drafts. All authors have critically reviewed and approved the final draft, and are responsible for the content and similarity index of the manuscript.
